# Ocular manifestations of monkeypox: a case report

**DOI:** 10.5935/0004-2749.2022-0281

**Published:** 2025-02-11

**Authors:** Pedro Antonio Nogueira Filho, Carolina Dos Santos Lazari, Celso Francisco Hernandes Granato, Marina Akiko Rampazzo Del Valhe Shiroma, Aline Lopes Dos Santos, Mauro Silveira De Queiroz Campos, Denise Freitas

**Affiliations:** 1 PEnsE, Research, Teaching and Experimentation, Vision One; 2 Department of Ophthalmology and Visual Sciences, Escola Paulista de Medicina, Hospital São Paulo, Universidade Federal de São Paulo, São Paulo, Brazil; 3 Fleury Diagnostic Medicine, São Paulo, Brazil; 4 Department of Medicine, Discipline of Infectology, Escola Paulista de Medicina, Hospital São Paulo, Universidade Federal de São Paulo, São Paulo, Brazil

**Keywords:** Monkeypox, Monkeypox virus, Orthopoxvirus, Eye manifestations, Conjunctivitis, Varíola dos macacos, Vírus da varíola dos macacos, Orthopoxvirus, Manifestações oculares, Conjuntivite

## Abstract

Monkeypox disease is a viral zoonosis with symptoms similar to those seen in the past in smallpox (variola), although clinically less severe. Following the eradication of smallpox in 1980 and the subsequent cessation of smallpox vaccination, monkeypox has emerged as the most important orthopoxvirus from a public health standpoint. Monkeypox virus occurs primarily in central and western Africa, often in tropical forests, and has increasingly manifested in urban areas. Animal hosts include various rodents and nonhuman primates. We report the case of a patient with monkeypox disease who developed ocular complaints (eye discomfort and conjunctivitis) and had detectable conjunctival lesions on biomicroscopy and fluorescein testing. Its ophthalmological manifestations are still poorly known.

## Abstract (pt)

Varíola do Macaco é uma zoonose viral com sintomas semelhantes aos observados no passado em pacientes com Varíola, embora seja clinicamente menos grave. Com a erradicação da varíola em 1980 e a subsequente cessação da vacinação contra a varíola, a varíola dos macacos emergiu como o ortopoxvírus mais importante em saúde pública. O vírus monkeypox ocorre principalmente na África central e ocidental, muitas vezes nas proximidades de florestas tropicais, e tem se manifestado cada vez mais em áreas urbanas. Os hospedeiros animais incluem uma variedade de roedores e primatas não humanos. O presente estudo relata o caso de um paciente com Monkeypox que evoluiu com queixa oftalmológica de desconforto ocular e conjuntivite e, à biomicroscopia e teste da fluoresceína, detecção de lesões conjuntivais. Alterações oftalmológicas da doença são, ainda, pouco conhecidas.

## INTRODUCTION

*Orthopoxvirus* (family Poxviridae) encompasses variola (smallpox) virus, vaccinia virus, monkeypox virus (MPXV), and cowpox virus. In 1980, the World Health Organization (WHO) declared the global eradication of smallpox. Since the 1970s, human cases of monkeypox have been reported in several African countries^(1)^. From 1996 to 1997, an outbreak was reported in the Democratic Republic of the Congo. Nigeria also experienced outbreaks from 2017 to 2019, with a case fatality rate of approximately 3%. These outbreaks in Nigeria and the one in Cameroon in 2018 occurred in locations where monkeypox had not been reported for over 20 years.

Monkeypox is global public health concern, as it affects not only West and Central African countries, but the world. In 2003, the first monkeypox outbreak outside Africa occurred in the USA, and it was related to contact with infected pet prairie dogs. Monkeypox has also been reported in travelers from Nigeria to Israel and the UK (September 2018), Singapore (May 2019), and USA (July and November 2021). In May 2022, several monkeypox cases were identified in several non-endemic countries where no previous outbreaks had been reported^(2)^.

An international case series describing the clinical course of patients with polymerase chain reaction (PCR)- confirmed MPXV infection found that 98% were gay or bisexual men, 75% were white, and 41% had comorbid human immunodeficiency virus infection, and the median age was 38 years. In 95% of cases, transmission was suspected to have occurred through sexual activity. In this case series, 95% of the patients had a rash (64% had <10 lesions), 73% had anogenital lesions, and 41% had mucosal lesions (54 had a single genital lesion). Common systemic features that precede the rash include fever (62%), lethargy (41%), myalgia (31%), and headache (27%). Lymphadenopathy was also common (56%). MPXV DNA was detected in 29 of the 32 patients in which seminal fluid was analyzed. Antiviral treatment was given to 5% of patients overall, and 70 (13%) were hospitalized. The reasons for hospitalization were pain management, mainly due to severe anorectal pain (n=21), soft tissue superinfection (n=18), pharyngitis limiting oral intake (n=5), ocular lesions (n=2), acute kidney injury (n=2), myocarditis (n=2), and infection control purposes (n=13). No deaths were reported^(3)^.

Signs and symptoms generally last 2-4 weeks. The incubation period (during which the infected person is asymptomatic) is typically 6-16 days, but can be as long as 21 days. Initial symptoms include sudden onset of fever, headache, muscle aches, back pain, lymphadenopathy, chills, and exhaustion.

In the literature, the ocular manifestations most often described are enlarged lymph nodes (including preauricular lymph nodes), vesicular blepharitis, conjunctival skin lesions, focal conjunctivitis, and corneal ulcers, etc.

## CASE REPORT

**Figure 1:**
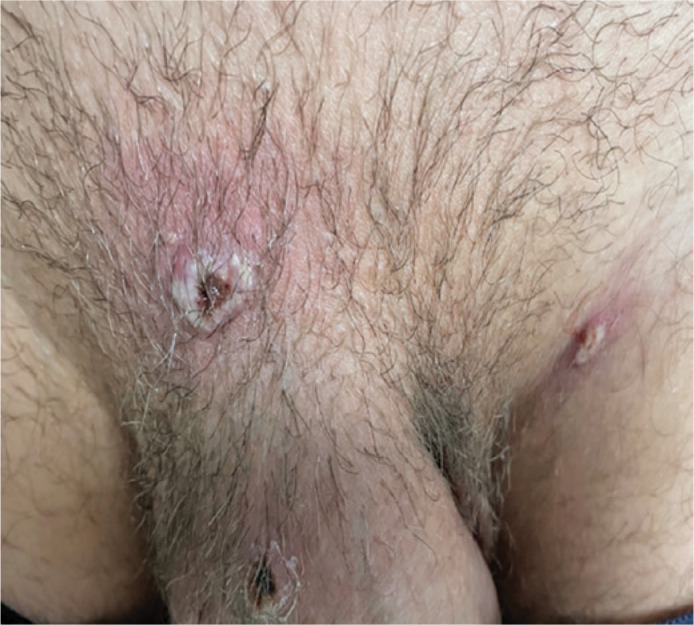
Skin lesions, similar to pustules and 03 in number, in the pelvic region “in a man with monkeypox disease” confirmed by polymerase chain reaction from the respective collection of microbiological materials from the wounds.

A 30-year-old man presented with a 1-day history of pruritus, foreign-body sensation (“sand”), and photophobia in his right eye (RE). He denied pain or impaired visual acuity. He reported a flu-like malaise that had preceded the onset of eye symptoms by approximately 5 days and was associated with episodes of diffuse myalgia, which he described as mild and low-grade fever (average temperatures of 37.5°C during the first 4 days of symptoms). He also reported whole-body pruritus, especially in the inguinal region bilaterally, where at least three cutaneous lesions (Figure 1) had developed approximately 5 days before his ophthalmologic consultation. Swabs taken from vesicular skin lesions were positive for MPXV in the PCR with a cycle threshold (Ct) value of 15.

**Figure 2:**
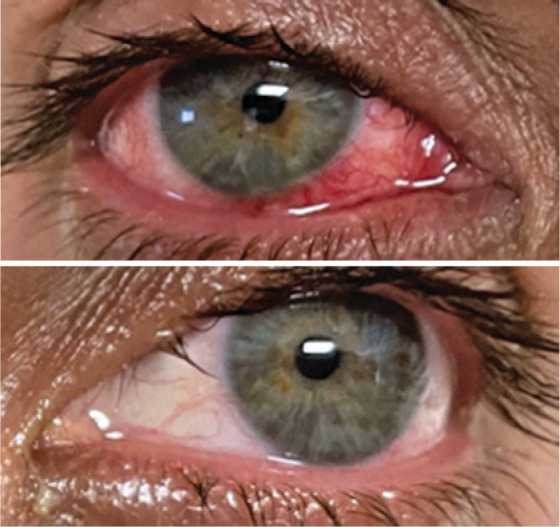
Anterior segment/biomicroscopy showing the presence of hyaline secretion on the ocular surface, hyperemia, and conjunctival vascular congestion with emphasis on the lower nasal quadrant “in the right eye (RE) of a man with monkeypox confirmed by polymerase chain reaction,” from the respective collection of microbiological material from the ocular surface of the RE.

**Figure 3:**
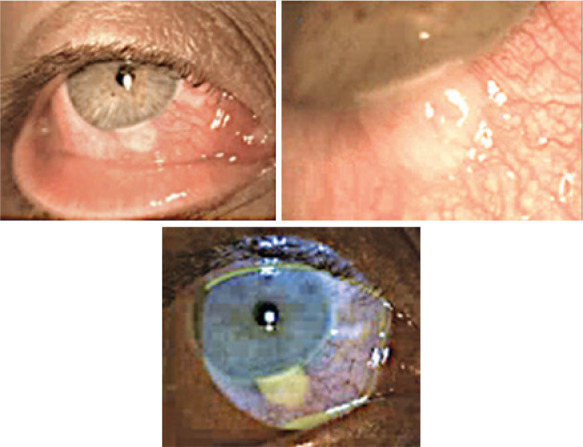
Anterior segment/biomicroscopy showing the presence of discreet follicles in the middle and temporal thirds of the lower tarsal conjunctiva, about 03 ulcerated epithelial conjunctival lesions, on the caruncle, nasal equator between the caruncle and the limbus, and limbar region in the inferior nasal quadrant of the cornea of the right eye (RE), measuring approximately 3 × 3 mm each, with a flat surface and covered by fibrotic material of milky color “in the right eye (RE) of a man with monkeypox confirmed by polymerase chain reaction,” from the respective collection of microbiological material from the ocular surface of this eye.

On ophthalmologic examination, the visual acuity in both eyes was 20/20. On external examination, pitting edema of the upper eyelid and diffuse ocular hyperemia were observed. No lymphadenopathy was identified on cervical and preauricular palpation. Extraocular motility was within the normal limits. Slit-lamp biomicroscopy showed copious watery discharge on the ocular surface, hyperemia and mild conjunctival vascular congestion, discrete follicles in the middle and temporal thirds of the lower tarsal conjunctiva, and three ulcerated epithelial conjunctival lesions (on the caruncle, nasal equator between the caruncle and limbus, and limbal region of the inferior nasal quadrant of the RE cornea), which measured approximately 3 × 3 mm each, with a flat surface and covered by milky white fibrotic material (Figures 2 and 3).

The cornea was spared, as were the anterior and posterior segments of the eye, including the retina. The left eye was completely normal. Samples taken from the conjunctival lesions were also positive for MPXV in the PCR (Ct=15). The therapeutic approach consisted only of symptomatic drugs of systemic use for febrile episodes (500 mg dipyrone monohydrate every 6/6 h) in addition to the topical use of preservative-free lubricating eye drops (0.15% sodium hyaluronate every 3/3 h) and as topical prophylaxis (tobramycin 0.3% eye drops every 8 h for 10 days). The patient is followed up weekly and is still recovering from the residual ocular inflammatory condition.

## DISCUSSION

Available evidence suggests that several ocular manifestations are associated with MPXV infection, given the current frequency of this disease and the fact that it has been declared by the WHO as a public health emergency of international concern.

The systemic clinical picture of MPXV infection is very similar to that of common and modified-type smallpox, with an incubation period ranging from 5 to 21 days^(4-6)^. Lymphadenopathy occurs in early disease stages and is a hallmark of monkeypox, which differentiates it from smallpox and chickenpox^(7-9)^. Despite ocular involvement, the patient did not present with palpable preauricular or submandibular lymphadenopathy at any of his three visits to date. Inguinal lymphadenopathy was reported by the patient, who is a physician. Importantly, MPXV causes lymphadenopathy, which may involve the preauricular lymph nodes, as seen in viral conjunctivitis^(5-7)^.

The cutaneous lesions characteristic of MPXV usually progress from macular, to papular, to vesicular, and then pustular^(7)^, which may involve the periorbital and orbital skin. However, in this case, palpebral cutaneous involvement was not observed, which strongly suggests that both conjunctivitis and conjunctival lesions were not caused by contiguous spread, but possibly through the hematogenous route.

In the literature, conjunctivitis and eyelid edema have been described in approximately 20% of the patients and resulted in additional physical and mental distress, albeit transient^(5,7)^.

Interestingly, Jezek et al. showed that conjunctivitis was more common among patients affected by animalacquired MPXV (20.3%) compared with those affected by human-to-human spread (16.4%)^(8)^. In addition, focal lesions on the conjunctiva and along the eyelid margins were seen with a higher incidence among patients unvaccinated for MPXV (68/294, approximately 25%)^(5)^. As in the case described herein, conjunctival lesions were identified, and the patient was not vaccinated for smallpox^(9)^. Importantly, smallpox vaccination, which was conducted until the disease was eradicated in the 1980s, may provide some levels of protection against monkeypox. Hughes et al. reported that patients in whom ocular involvement was observed had a higher frequency of other ocular symptoms, such as photophobia, as well as systemic symptoms such as nausea, chills, sweating, oral ulcers, sore throat, malaise, and lymphadenopathy. Conjunctivitis may be predictive of the disease course because 47% of the patients with conjunctivitis reported systemic involvement compared with 16% of the patients without ocular involvement^(6)^.

Photophobia alone, without ocular involvement, was reported in approximately 22% of the patients^(7)^. In addition, infection can result in severe keratitis (7.5% of the patients in one study) and corneal scarring (4% of patients without vaccination and 1% of previously smallpox-vaccinated cases), potentially leading to permanent vision loss^(6-8)^. Jezek et al.^(8)^ observed unilateral or bilateral blindness and impaired vision in 10% of the primary cases (infection from an animal source) and 5% of the secondary cases (the rash appeared 721 days after exposure to another human case, potentially reflecting person-to-person transmission). Frontal headache involving the orbits has also been reported^(5,6)^. In another study, blepharitis was observed in 30% of the patients without vaccination and 7% of those with smallpox vaccination.

According to the WHO, fluid samples collected from pustules or dry crusts from scaly lesions are optimal for diagnostic purposes. Lesion biopsy specimens can also be used. However, blood samples are not recommended because the virus remains in the bloodstream only briefly during the infection.

Monkeypox is usually a self-limiting disease, with symptoms lasting 2-4 weeks. Clinical diagnosis can be challenging, as MPXV infection can present with various manifestations, including ocular ones.

Most ophthalmic manifestations associated with MPXV are more common than the assumption of a rare event (<5%), given the continuous, rapid, and significant increase in the number of MPXV infection cases and patients presenting with conjunctivitis, blepharitis, keratitis, or corneal and conjunctival lesions.

PCR can detect a virus present in a sample taken from any such ocular lesions, revealing whether the patient has active infection during the test. Given the sensitivity and precision of this technique, PCR is the preferred laboratory test for the diagnosis of monkeypox.

Notably, severe sequelae and complications of monkeypox occur more commonly among unvaccinated populations (74%) compared with vaccinated ones (39.5%)^(5,6)^. Thus, there is a need to reintroduce the administration of smallpox vaccines to high risk groups.

Regarding ophthalmologic treatment, highlighting the potential benefits of relatively simple therapies for ocular manifestations, such as lubricants or topical antibiotics, is important. The antiviral agent cidofovir may also be effective against MPXV and can be indicated in severe systemic cases and ocular manifestations with high risk of visual loss. In the latter cases, topical use of trifluoridine is possible^(9,10)^.
